# Editorial: Structures, Signaling Mechanisms, and Functions of Types I and III Interferons

**DOI:** 10.3389/fimmu.2021.638479

**Published:** 2021-02-18

**Authors:** Ronald L. Rabin, Mark R. Walter

**Affiliations:** ^1^Center for Biologics Evaluation and Research, U.S. Food and Drug Aministration (USFDA), Silver Spring, MD, United States; ^2^University of Alabama at Birmingham, Birmingham, AL, United States

**Keywords:** interferon α, interferon λ, interferon subtypes, interferon receptors, interferon structure, interferon signaling, interferon function, interferon therapy

Never has understanding the fundamental roles of the interferons (IFNs) been more important than in the year 2021. It is well-recognized that IFNs play critical roles inducing an antiviral state in cells. However, their influence on innate and adaptive immunity continues to expand. As a result, IFNs play critical roles in protecting the host from pathogens, controlling cellular transformation, and when dysregulated, promoting autoimmunity. Since the seminal discovery of Type I IFNs over 60 years ago (1), the Type I and Type III IFN family has grown to include 20 distinct members consisting of 16 Type I IFNs (12 IFNαs, IFNβ, IFNε, IFNκ, and IFNω) and four Type III IFNs (IFNλ1-IFNλ4) (2) that signal through common type-I (IFNAR1/IFNAR2) and type-III IFNλR1/IL10R2 receptor complexes. The paucity of studies that define the role of the IFN subtypes (Type I/Type III) in cellular function and disease was a major driver of this research topic.

“Structures, Signaling Mechanisms, and Functions of Types I and III Interferons” is a collection of eight review articles that are intended to summarize current knowledge on fundamental aspects of interferon signaling and biology. Three articles address IFN receptor biology and signaling. Walter compares the structures of types I, II, and III IFNs and their receptor complexes, providing insights into how subtle structural differences in the IFNs may modulate downstream signaling. Notably, the study highlights murine IFNβ does not share equivalent structural and biophysical properties with its human counterpart, highlighting the difficulties in direct comparisons of type-I IFN signaling between species. Subsequently, Zanin et al. reviews type I IFN receptor trafficking. These authors comprehensively discuss post-translational modification of the type I IFNAR receptors and the role of clathrin-mediated endocytosis on signaling, recycling, or degradation of receptor components. Mazewski et al. review canonical and non-canonical signaling mechanisms, and discuss their roles in infectious and autoimmune diseases, and in cancer. Ultimately, the outcome of IFN signaling is the production of various levels of IFN stimulated genes (ISGs). Thus, Yang and Li provide a detailed review of the complex anti-viral defense mechanisms used by a small set of ISGs to inhibit viral RNA replication.

Three additional articles review our current understanding of the unique and overlapping roles of the type-I and type-III IFNs. Stanifer et al. discuss redundant and non-redundant expression patterns, signaling, and functional outcomes between types I and III IFN at respiratory and intestinal barriers. Notably, this work highlights the role of epithelial cell heterogeneity and cell polarity in explaining the non-redundant activities of type III IFNs at barrier surfaces. Two articles focus on functional differences among different type I IFN subtypes. Fox et al. provide an extensive review highlighting differences among IFNα and IFNβ subtypes in murine infectious disease models, with potential insights for human disease. Wittling et al. review human type I IFNs to propose that regulatory elements, expression patterns, and primate evolution can serve as a guide toward revealing unique roles for the IFNα subtypes. These reviews, as well as Mazewski et al. discuss various aspects of using type I IFNs to treat patients with chronic autoimmune and infectious diseases. As expected, the potential of type I IFN as a therapy for SARS-CoV-2 is discussed in several reviews. In particular, the review by Schreiber highlights the roles for type I IFNs in viral defense and as a therapeutic agent to ameliorate the ongoing pandemic caused by the virus.

We wish to extend our extreme gratitude toward our colleagues who contributed to this review series as well as to those who took the time to review these manuscripts under the challenges of a pandemic. We hope this research topic summarizes the field for experienced biologists, serves as a foundation for neophytes entering the field, and stimulates novel research directions to harness the power of the IFNs to treat human disease.

## Author Contributions

All authors listed have made a substantial, direct and intellectual contribution to the work, and approved it for publication.

## Conflict of Interest

The authors declare that the research was conducted in the absence of any commercial or financial relationships that could be construed as a potential conflict of interest.
